# ECG T‐Wave Morphologic Variations Predict Ventricular Arrhythmic Risk in Low‐ and Moderate‐Risk Populations

**DOI:** 10.1161/JAHA.121.025897

**Published:** 2022-08-29

**Authors:** Julia Ramírez, Antti Kiviniemi, Stefan van Duijvenboden, Andrew Tinker, Pier D. Lambiase, Juhani Junttila, Juha S. Perkiömäki, Heikki V. Huikuri, Michele Orini, Patricia B. Munroe

**Affiliations:** ^1^ Clinical Pharmacology and Precision Medicine William Harvey Research Institute, Barts and The London School of Medicine and Dentistry, Queen Mary University of London London United Kingdom; ^2^ Aragon Institute of Engineering Research University of Zaragoza Zaragoza Spain; ^3^ Centro de Investigación Biomédica en Red ‐ Bioingeniería, Biomateriales y Nanomedicina Zaragoza Spain; ^4^ Research Unit of Internal Medicine Medical Research Center Oulu, University of Oulu and Oulu University Hospital Oulu Finland; ^5^ Institute of Cardiovascular Science University College London London United Kingdom; ^6^ National Institute for Health and Care Research Barts Cardiovascular Biomedical Research Centre Barts and The London School of Medicine and Dentistry, Queen Mary University of London London United Kingdom; ^7^ Barts Heart Centre St Bartholomew’s Hospital London United Kingdom

**Keywords:** ECG, noninvasive risk prediction, sudden cardiac death, T‐wave morphology, Electrophysiology, Sudden Cardiac Death, Arrhythmias, Ventricular Fibrillation

## Abstract

**Background:**

Early identification of individuals at risk of sudden cardiac death (SCD) remains a major challenge. The ECG is a simple, common test, with potential for large‐scale application. We developed and tested the predictive value of a novel index quantifying T‐wave morphologic variations with respect to a normal reference (TMV), which only requires one beat and a single‐lead ECG.

**Methods and Results:**

We obtained reference T‐wave morphologies from 23 962 participants in the UK Biobank study. With Cox models, we determined the association between TMV and life‐threatening ventricular arrhythmia in an independent data set from UK Biobank study without a history of cardiovascular events (N=51 794; median follow‐up of 122 months) and SCD in patients with coronary artery disease from ARTEMIS (N=1872; median follow‐up of 60 months). In UK Biobank study, 220 (0.4%) individuals developed life‐threatening ventricular arrhythmias. TMV was significantly associated with life‐threatening ventricular arrhythmias (hazard ratio [HR] of 1.13 per SD increase [95% CI, 1.03–1.24]; *P*=0.009). In ARTEMIS, 34 (1.8%) individuals reached the primary end point. Patients with TMV ≥5 had an HR for SCD of 2.86 (95% CI, 1.40–5.84; *P*=0.004) with respect to those with TMV <5, independently from QRS duration, corrected QT interval, and left ventricular ejection fraction. TMV was not significantly associated with death from a cause other than SCD.

**Conclusions:**

TMV identifies individuals at life‐threatening ventricular arrhythmia and SCD risk using a single‐beat single‐lead ECG, enabling inexpensive, quick, and safe risk assessment in large populations.

**Nonstandard Abbreviations and Acronyms**
CCS: Canadian Cardiovascular Society
LTVA: life‐threatening ventricular arrhythmia
MACE: major adverse cardiovascular event
non‐SCD: death from a cause other than SCD
QTc: corrected QT
SCD: sudden cardiac death
TMR: T‐wave morphologic restitution
TMV: T‐wave morphologic variations with respect to a normal reference
Tpe: T‐peak–to–T‐end

**Clinical Perspective**

**What Is New?**

- A novel index, T‐wave morphologic variations with respect to a normal reference, quantifies abnormal T‐wave morphologic variations from a single beat on a single‐lead ECG.
- T‐wave morphologic variations with respect to a normal reference is the only ECG marker associated with life‐threatening ventricular arrhythmias in individuals without cardiovascular disease, and it is strongly associated with sudden cardiac death in patients with coronary artery disease independently from QT interval and left ventricular ejection fraction.
- T‐wave morphologic variations with respect to a normal reference is not associated with death from a cause other than sudden cardiac death.

**What Are the Clinical Implications?**

- This is the first study evaluating T‐wave morphologic variations with respect to a normal reference; this novel index has the potential for predicting sudden cardiac death when measured from wearables in large‐scale screening.

Sudden cardiac death (SCD) is a leading cause of mortality, responsible for approximately half of all cardiovascular deaths.^1^ An effective and easily measured tool to identify individuals at risk within the general population is lacking.^2^ From a public health perspective, such a simple and easily available marker would permit identification of individuals to prioritize for monitoring and intervention and could be embedded in wearable devices.

Life‐threatening ventricular arrhythmias (LTVAs) are an important cause of morbidity and SCD in almost all forms of heart disease,^3^ and one of the main contributors to LTVAs leading to SCD is an enhanced spatiotemporal dispersion of ventricular repolarization.^4^ The surface ECG is a low‐cost, widely available, noninvasive tool, and it is considered a potential candidate for rapid risk assessment of LTVA and SCD. Because dispersion of ventricular repolarization is reflected in the ECG T‐wave,^5^ several single‐lead T‐wave indexes have been proposed as SCD predictors, including T‐wave inversions,^6^ the T‐peak–to–T‐end (Tpe) interval,^7^ early repolarization pattern,^8^ or the corrected QT (QTc) interval, but none has shown to be an effective risk predictor, potentially because they do not capture the overall T‐wave morphologic information.^9^, ^10^ An effective SCD risk predictor that could be easily measured from a single beat and a single lead is needed for translation to large‐scale screening and potential clinical application.

At a population level, deviations of traditional T‐wave indexes, like the QTc interval or the Tpe interval, from standard thresholds measured from resting ECGs indicate increased cardiovascular risk.^11^, ^12^ We therefore hypothesized that the T‐wave morphologic variations with respect to a normal reference (TMV) index, quantifying overall T‐wave morphologic variations with respect to a normal reference, from a single beat from a standard ECG single lead, could be a stronger marker for SCD risk stratification (Figure 1). In this work, we propose and develop an algorithm to calculate TMV. Then, we test its predictive value for LTVA in a large cohort of middle‐aged volunteers with no history of cardiovascular events and for SCD in a cohort of patients with coronary artery disease.

**Figure 1:**
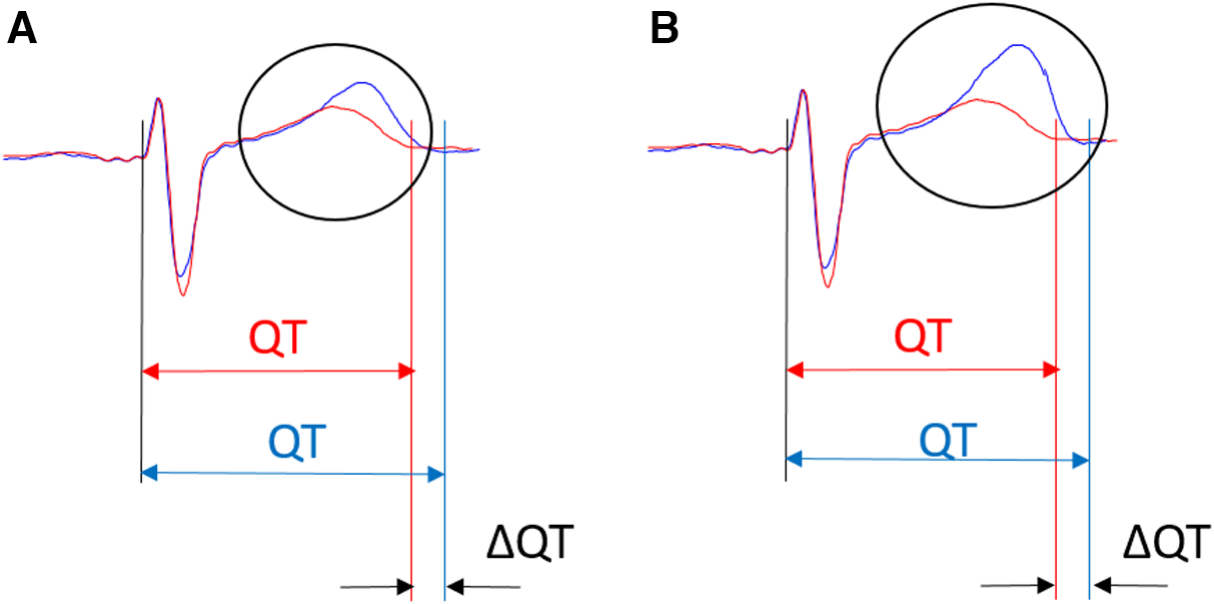
Main hypothesis of this work: T‐wave morphologic variations with respect to a normal reference (TMV) can occur with same QT interval values. **A**, An example where an individual has a T‐wave morphology (blue) with low deviations from a normal reference (red), leading to low changes in the QT interval and low values of TMV, which is proposed and tested in this work. **B**, An example where an individual has a T‐wave morphology (blue) with larger variations with respect to the normal reference (red), leading to larger TMV values, despite showing low changes in the QT interval.

## METHODS

Anonymized data and materials have been returned to UK Biobank and can be accessed per request.

### Reference Cohort

**Figure 2:**
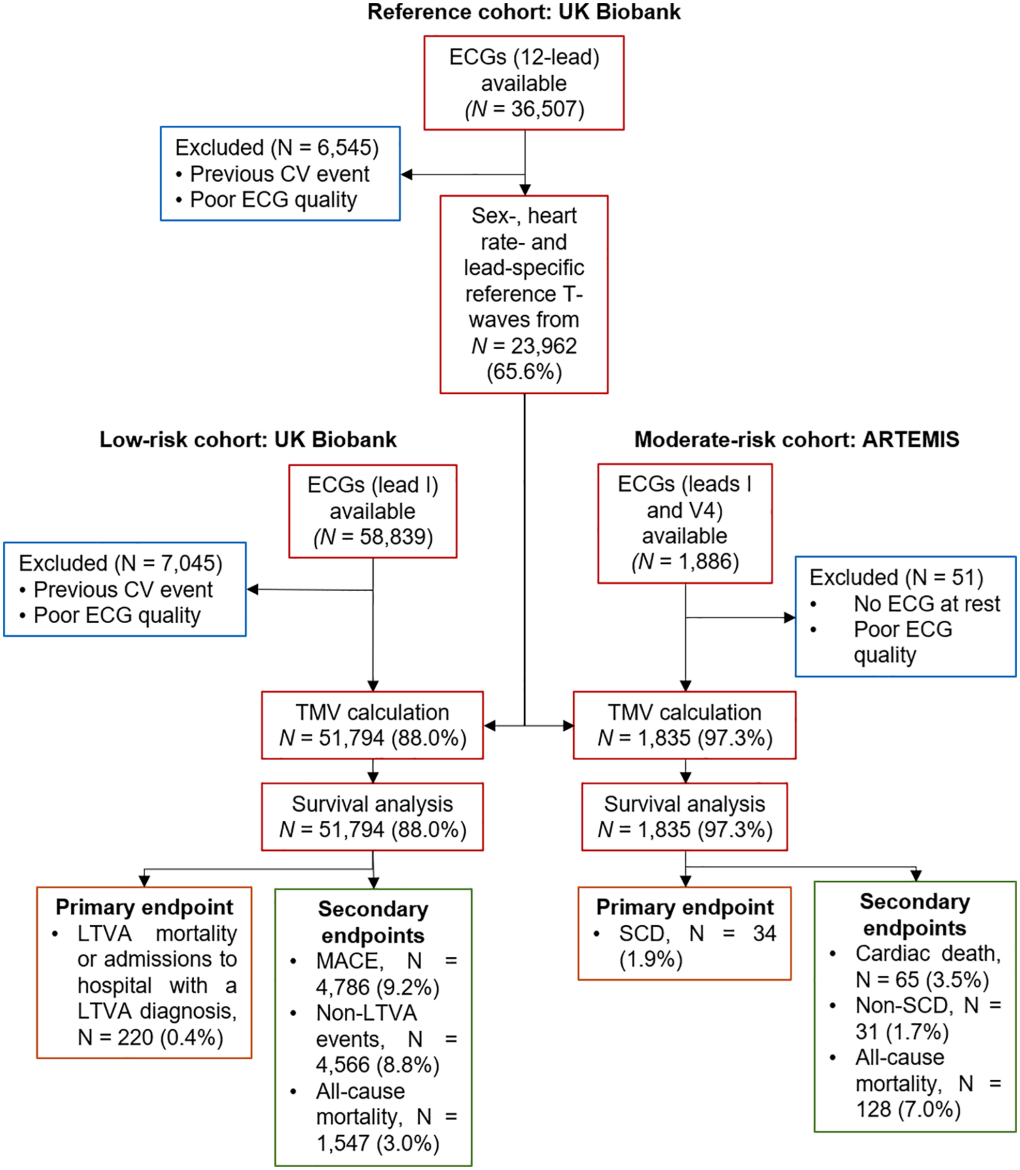
Flow diagram of the study design. Sex‐, heart rate–, and lead‐specific T‐waves are obtained from a reference cohort in UK Biobank. The T‐wave morphologic variations with respect to a normal reference (TMV) index is calculated comparing the T‐wave morphology deviation of T‐waves in a low‐risk population (UK Biobank) and in a moderate‐risk population (ARTEMIS) from the reference T‐waves. The risk stratification value of TMV is tested in survival analyses. CV indicates cardiovascular; LTVA, life‐threatening ventricular arrhythmia; MACE, major adverse cardiovascular event; Non‐SCD, death from a cause other than SCD; and SCD, sudden cardiac death.

Sex‐, heart rate–, and lead‐specific normal T‐wave morphologic references were calculated from standard 10‐second, 12‐lead ECG recordings at rest in a population of 23 962 middle‐aged men and women without a history of cardiovascular events from the UK Biobank (reference cohort; Figure 2 and Data S1; UK Biobank application 8256; the study was approved by an institutional review committee, and all subjects gave informed consent).

### Low‐Risk Test Cohort, UK Biobank

For the low‐risk test cohort, we selected 58 839 individuals from an independent cohort within UK Biobank who participated in an exercise stress test. These individuals were not part of the reference cohort (Figure 2 and Data S1). All individuals in this cohort had a 15‐second resting ECG recorded before exercise stress test. Only lead I was recorded. Individuals were excluded if they had a previous cardiovascular event (matching the codes from Table S1) or if the ECG had poor quality, leaving 51 794 individuals included in the analyses.

The primary end point for this cohort was LTVA, defined as LTVA mortality or admission to hospital with an LTVA diagnosis. Definitions and codes are provided in Table S1. The secondary end points were major cardiovascular events (MACEs; including mortality or admissions to hospital; Table S1). Follow‐up was from the study inclusion date until June 22, 2020.

### Moderate‐Risk Test Cohort, ARTEMIS

A period of 15‐second resting ECG was analyzed for 1886 patients from Finland with coronary artery disease (leads I and V4) from the ARTEMIS study.^13^ Fifty‐one subjects were excluded because of no ECG at rest or poor ECG quality, leading to 1835 individuals included in the analyses (Figure 2). All enrolled patients gave informed consent, and the institutional ethics committee approved the study. The study complies with the Declaration of Helsinki.

The primary end point was SCD or resuscitation from sudden cardiac arrest, whichever occurred first. The definition for SCD was a witnessed death within 1 hour of the onset of symptoms. For unwitnessed deaths, the definition was last being seen alive and stable 24 hours before discovery. The secondary end points were cardiac death (including SCD, aborted sudden cardiac arrest, and death from a cause other than SCD [non‐SCD], whichever occurred first), non‐SCD, and all‐cause mortality. Follow‐up was 5 years.^13^

### TMV Index, T‐Wave Morphologic Variations With Respect to a Normal Reference

**Figure 3:**
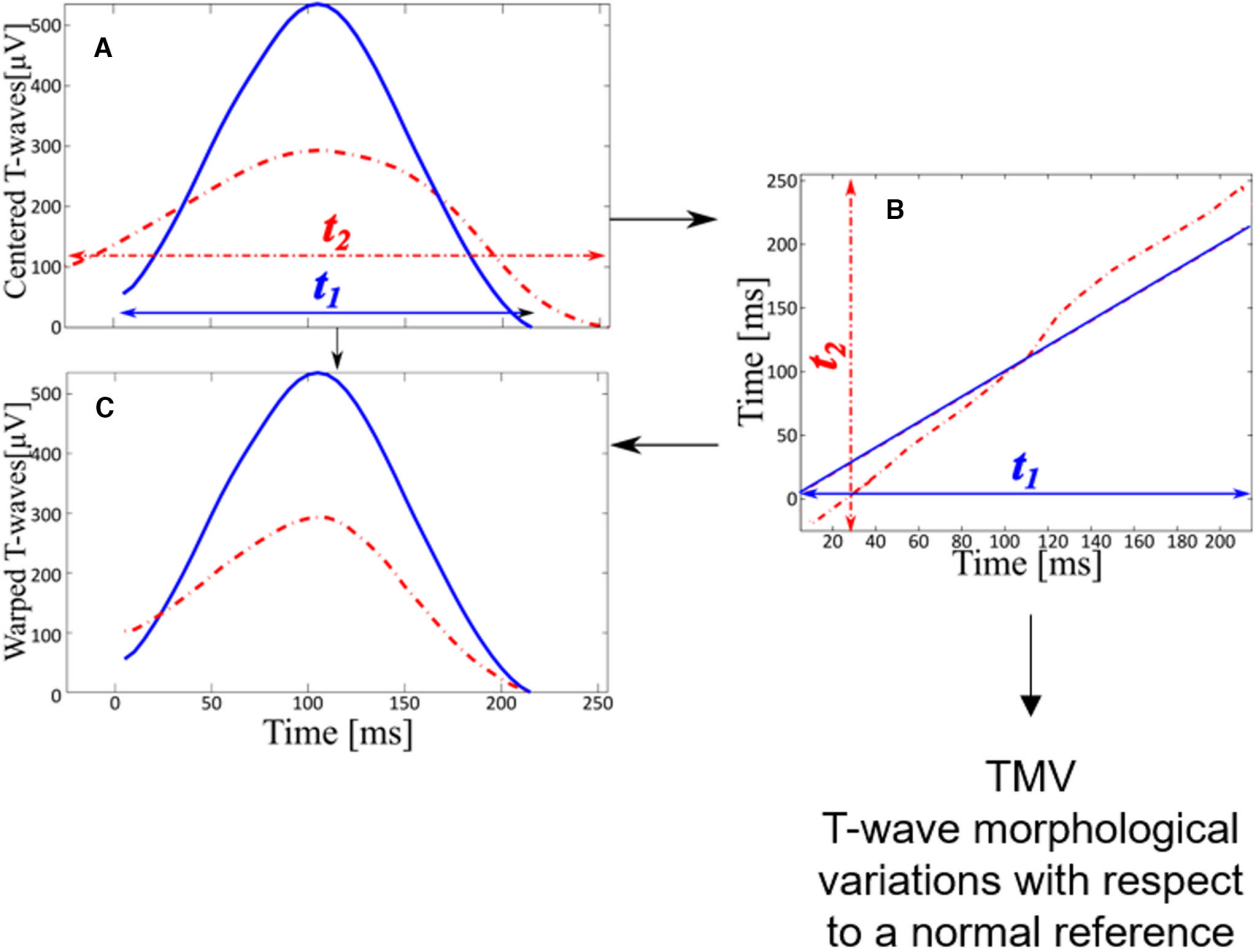
Quantification of T‐wave morphologic variations with respect to a normal reference (TMV). **A**, A normal T‐wave reference (blue) and an average T‐wave from an individual participant (red) are prealigned with respect to their gravity centers. **B**, Dynamic programming is applied to find the warping function (red) that optimally aligns (warps) in time both T‐waves. **C**, TMV is calculated as the average deviation of the warping function (**B**) from the diagonal.

In the low‐risk cohort (UK Biobank), we calculated TMV by comparing the average T‐wave from each participant with his/her corresponding sex and RR normal T‐wave morphologic reference in lead I from the reference cohort (Figure 2). In short, we used our previously published algorithm based on dynamic time warping^14^ to derive TMV, quantifying the average temporal stretching necessary to align each point of the reference T‐wave to the average T‐wave from UK Biobank (Figure 3). The specific equation of TMV is as follows:

(1) $$\mathrm{TMV}=\frac{1}{N_{r}}\sum_{n=1}^{N_{r}}\left|\gamma \times \left(t^{r}\left(n\right)\right)\cdot \frac{f^{r}\left(t^{r}\left(n\right)\right)}{\max\left(f^{r}\left(t^{r}\left(n\right)\right)\right)}-t^{r}\left(n\right)\right|$$

where $\gamma \times \left(t^{r}\right)$ is the optimal warping function relating the average T‐wave from each participant to its corresponding sex and RR normal T‐wave morphologic reference ($f^{r}\left(t^{r}\right)$, of length $N_{r}$), with an additional weighted variable that has recently proved to be more robust against noise.^15^

We, then, followed the same procedure to derive TMV in the moderate‐risk cohort (ARTEMIS) from lead I (to ease comparisons across cohorts) and from lead V4 (optimal to capture ventricular repolarization as it usually shows the T‐wave with the highest energy, but not available in UK Biobank). The derivation of TMV and its association with events in ARTEMIS were performed in a blinded manner.

### Statistical Analysis

In UK Biobank, the QT and Tpe intervals were measured as the intervals between the QRS onset and the T‐wave end, and between the T‐wave peak and the T‐wave end, respectively, from the averaged heartbeat at rest. Then, we corrected the QT interval using Bazett formula.^16^ We additionally derived the marker T‐wave inversion, which indicated a change in the polarity of the T‐waves,^6^ and the QRS duration. In ARTEMIS, these ECG indexes were automatically derived using custom made software.^17^ Missing data were imputed using the “mice” package in R, provided a missing rate <10%. Variables with a higher rate of missingness were excluded.

The 2‐tailed Mann‐Whitney and Fisher exact tests were used for univariable comparison of quantitative and categorical data, respectively. The concordance index (C‐index) was calculated to estimate the performance of TMV in both UK Biobank and ARTEMIS. We estimated the optimal cutoff values for TMV in both low‐ and moderate‐risk cohorts based on the highest sum of specificity and sensitivity above median values with at least 20% sensitivity, as in previous studies.^10^ For these optimal cutoff values, we provide values of positive predictive value, negative predictive value, sensitivity, and specificity. Kaplan‐Meier curves were derived using the optimal cutoff values, with a comparison of cumulative events performed by using log‐rank tests, and plotted using the “survminer” package in R.

Univariable and multivariable Cox regression analyses were performed to determine the predictive value of the risk markers. Models were adjusted by risk factors shown in Table 1 (UK Biobank) and Table 2 (ARTEMIS). All continuous variables were standardized to a mean of 0 and SD of 1 to allow for comparisons in the Cox models. Only the variables with a significant association with the end point in univariable analysis were included in the multivariable model. Stepwise regression analysis was then performed to only retain the variables independently associated with the outcome. Individuals who died from causes not included in the primary end point were censored at the time of death. In ARTEMIS, TMV measured from leads I or V4 were entered one at a time into the multivariable model. Competing risks survival analyses (the Gray method)^18^ were also conducted using approaches of LTVA versus a non‐LTVA event in UK Biobank and SCD versus non‐SCD in ARTEMIS. The C‐index, as well as the net reclassification improvement index and the integrated discrimination improvement index, was calculated to estimate the improvement of adding the strongest TMV index (measured on lead I or on lead V4). A value of *P*<0.05 was considered statistically significant. Statistical analyses were performed using R version 4.0.2.

**Table 1:** Association With LTVAs in UK Biobank

Risk factor	Univariable analysis		Multivariable analysis	
	Hazard ratio (95% CI)	*P* value	Hazard ratio (95% CI)	*P* value
Sex (male)*	3.066 (2.283–4.119)*	<0.001*	2.493 (1.840–3.379)*	<0.001*
Age (per 1 SD)*	1.976 (1.682–2.321)*	<0.001*	1.795 (1.521–2.119)*	<0.001*
Diabetes (yes)	1.064 (0.546–2.074)	0.854	…	…
BMI (per 1 SD)	1.156 (1.023–1.307)	0.020	…	…
SBP (per 1 SD)*	1.489 (1.312–1.691)*	<0.001*	1.188 (1.033–1.366)*	0.016*
DBP (per 1 SD)	1.131 (0.992–1.289)	0.066	…	…
Previous or current smoker (yes)	1.167 (0.895–1.521)	0.254	…	…
Glycated hemoglobin (per 1 SD)	1.128 (1.026–1.241)	0.013	…	…
Glucose (per 1 SD)	1.051 (0.944–1.170)	0.360	…	…
Cholesterol (per 1 SD)	0.948 (0.829–1.083)	0.434	…	…
LDL (per 1 SD)	0.991 (0.868–1.132)	0.899	…	…
HDL (per 1 SD)	0.779 (0.674–0.900)	<0.001	…	…
Triglycerides (per 1 SD)	1.152 (1.031–1.287)	0.013	…	…
Creatinine (per 1 SD)*	1.145 (1.108–1.184)*	<0.001*	1.124 (1.058–1.194)*	<0.001*
Albumin (per 1 SD)	0.906 (0.794–1.035)	0.145	…	…
Albumin/creatinine ratio (per 1 SD)	0.616 (0.532–0.713)	<0.001	…	…
RR interval (per 1 SD)	0.904 (0.788–1.037)	0.148	…	…
QRS duration (per 1 SD)	1.111 (0.973–1.268)	0.119	…*	…*
T‐wave inversion (yes)	15.603 (2.188–111.267)	0.006	…	…
Tpe interval (per 1 SD)	0.996 (0.872–1.138)	0.957	…	…
QTc interval (per 1 SD)	1.093 (0.960–1.244)	0.178	…	…
TMV (per 1 SD, lead I)*	1.186 (1.078–1.306)*	<0.001*	1.131 (1.032–1.240)*	0.009*

Ellipses indicates variables were not significantly independently associated with LTVA in the multivariable model; BMI, body mass index; DBP, diastolic blood pressure; HDL, high‐density lipoprotein; LDL, low‐density lipoprotein; LTVA, life‐threatening ventricular arrhythmia; QTc, corrected QT (using the Bazett formula); SBP, systolic blood pressure; TMV, T‐wave morphologic variations with respect to a normal reference; and Tpe, T‐peak–to–T‐end.

* Significant variables in the multivariable model.

**Table 2:** Univariable and Multivariable Association With SCD in ARTEMIS

Risk factor	Univariable analysis		Multivariable analysis	
	Hazard ratio (95% CI)	*P* value	Hazard ratio (95% CI)	*P* value
Sex (male)	1.520 (0.688–3.357)	0.300	…	…
Age (per 1 SD)	1.582 (1.090–2.296)	0.016	…	…
Diabetes (yes)	2.247 (1.125–4.488)	0.022	…	…
PCI (angioplasty) vs no revascularization (reference)	1.306 (0.433–3.934)	0.625	…	…
CABG vs no revascularization (reference)	3.247 (1.078–9.784)	0.036	…	…
CCS class ≥2*	2.927 (1.427–6.005)*	0.003*	2.253 (1.086–4.675)*	0.029*
LVEF (biplane 2D measurement) (per 1 SD)*	0.520 (0.415–0.652)*	<0.001*	0.636 (0.496–0.815)*	<0.001*
LV mass index (per 1 SD)	1.552 (1.197–2.011)	0.001	…	…
RR interval (per 1 SD)	0.685 (0.483–0.972)	0.034	…	…
QRS duration (per 1 SD)	1.390 (1.080–1.788)	0.01	…	…
T‐wave inversions (any vs none)*	4.102 (2.000–8.415)*	<0.001*	2.650 (1.222–5.745)*	0.014*
Tpe interval (per 1 SD)	0.925 (0.658–1.301)	0.654	…	…
QTc interval (per 1 SD)	1.770 (1.325–2.365)	<0.001	…	…
TMV in lead I (per 1 SD)	1.213 (0.955–1.539)	0.113	1.002 (0.744–1.351)	0.987
TMV in lead V4 (per 1 SD)	1.319 (1.093–1.593)	0.004	1.206 (0.946–1.537)	0.130
TMV in lead I ≥2.4*	3.757 (1.796–7.856)*	<0.001*	2.308 (1.072–4.968)*	0.032*
TMV in lead V4 ≥5.0*	4.420 (2.254–8.667)*	<0.001*	2.864 (1.404–5.841)*	0.004*

Ellipses indicates variables were not significantly associated with SCD in the multivariable model; 2D, 2 dimensional; CABG, coronary artery bypass grafting; CCS, Canadian Cardiovascular Society (classification for angina pectoris); LV, left ventricular; LVEF, LV ejection fraction; PCI, percutaneous coronary intervention; QTc, corrected QT (corrected with the Bazett formula); SCD, sudden cardiac death; TMV, T‐wave morphologic variations with respect to a normal reference; and Tpe, T‐peak–to–T‐end.

* Significant risk factors in the multivariable model.

## RESULTS

The derived heart rate– and lead‐specific reference T‐wave morphologies for women and men are shown in Figures S1 and S2, respectively.

### Predictive Value in the Low‐Risk Cohort

The low‐risk population consisted of 23 954 men, aged 40 to 73 years (median [interquartile range] of 58 [13] years) after exclusions. The demographic characteristics of this population are shown in Table S2. During the follow‐up, 220 (0.4%) individuals had an LTVA, 1591 (3.1%) had a MACE, 1371 (2.6%) had a non‐LTVA event, and 1547 (3.0%) died of any cause.

Compared with individuals who did not experience LTVA during the follow‐up, participants with LTVAs were older (*P*<0.001) and had higher body mass index (*P*=0.019), systolic blood pressure (*P*<0.001), diastolic blood pressure (*P*=0.018), glycated hemoglobin (*P*=0.005), triglycerides (*P*=0.010), creatinine (*P*<0.001), and TMV (*P*=0.003). In addition, they had lower high‐density lipoprotein and albumin/creatinine ratio (*P*<0.001). Finally, there were significantly more men (*P*<0.001; Table S3).

**Figure 4:**
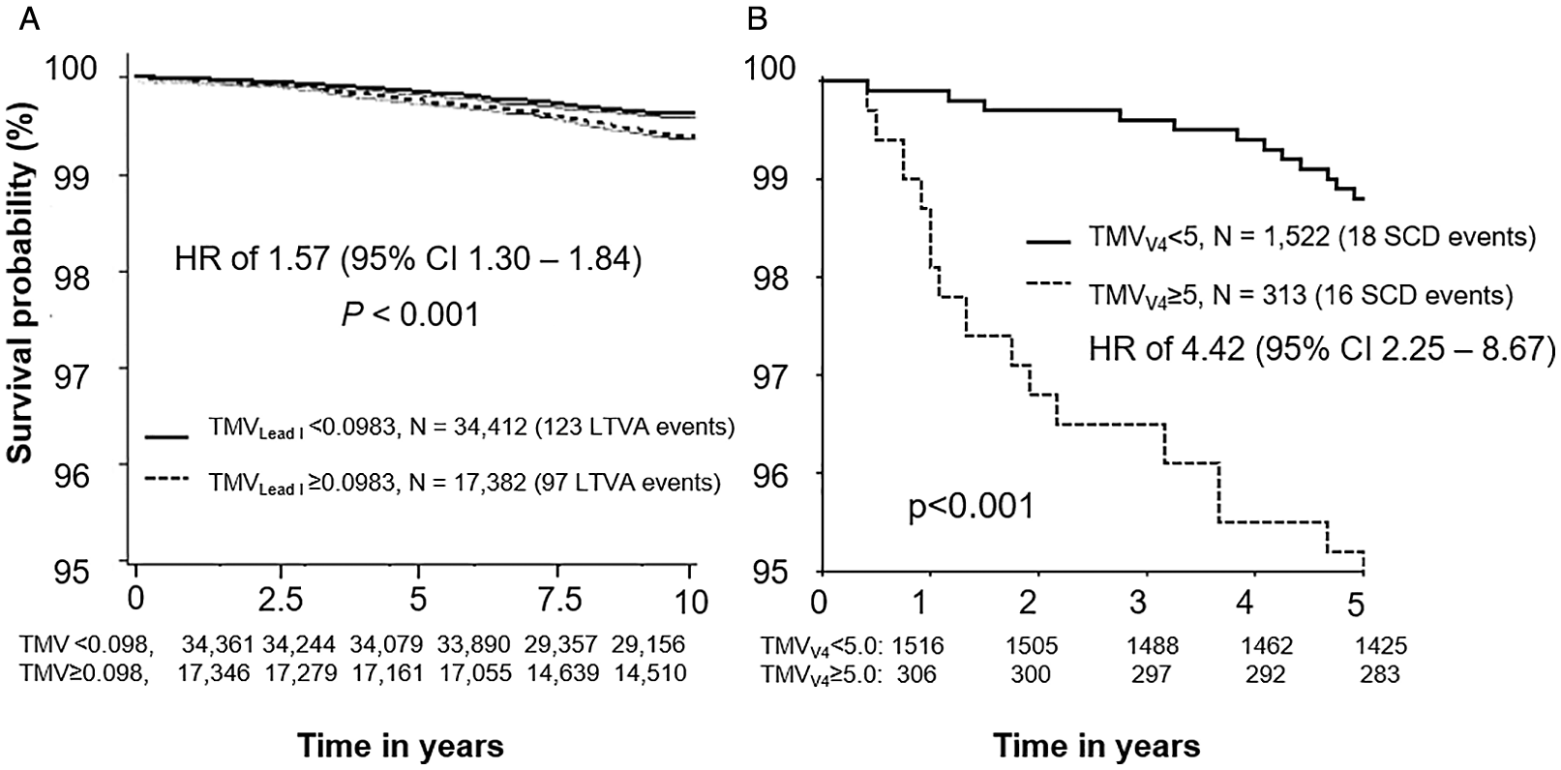
Cumulative survival rates of individuals stratified by TMV >0.0983 in the low‐risk cohort (UK Biobank; A) and by TMV >5 in the moderate‐risk cohort (ARTEMIS; B). Numbers below each graph represent the number of individuals at risk in each group. CI indicates confidence interval; HR, hazard ratio, LTVA, life‐threatening ventricular arrhythmia; SCD, sudden cardiac death; and TVA, T‐wave morphologic variations with respect to a normal reference.

The C‐index of TMV in UK Biobank was 0.558. The threshold TMV=0.0983 (stratified according to the optimal cutoff value in UK Biobank) showed a positive predictive value of 0.6%, a negative predictive value of 99.6%, sensitivity of 44.6%, and specificity of 66.5%. In univariable Cox analysis, participants with TMV ≥0.0983 had a hazard ratio (HR) of 1.57 (95% CI, 1.30–1.84) compared with participants with TMV <0.0983 (*P*<0.001; Figure 4A). In multivariable Cox analysis, the following variables remained significantly associated with LTVAs (HR [95% CI] reported): male sex (2.49 [1.84–3.38]), age (1.80 [1.52–2.12]), systolic blood pressure (1.19 [1.03–1.37]), creatinine (1.12 [1.06–1.19]), and TMV (1.13 [1.03–1.24]; Table 1). The C‐index of this model was 0.731. None of the other tested ECG markers (RR interval, QRS duration, T‐wave inversions, or Tpe or QTc interval) remained significantly associated with LTVAs. When adjusting for non‐LTVA as competing risk, we found the HRs for LTVA to be similar (Table S4).

In addition, in multivariable Cox analysis, TMV remained significantly associated with MACE and non‐LTVA events (HR [95% CI] of 1.06 [1.01–1.10] and of 1.05 [1.01–1.10], respectively), independently of age, male sex, diabetes, body mass index, systolic blood pressure, smoking status, glycated hemoglobin, glucose, low‐density lipoprotein, high‐density lipoprotein, creatinine, albumin, T‐wave inversions, and QTc interval (Tables S5 and S6). Finally, TMV was not independently associated with all‐cause mortality (Table S7).

### Predictive Value in a Moderate‐Risk Population

The ARTEMIS population consisted of 1835 individuals (1257 men; median [interquartile range] age of 67 [12] years) after exclusions. The demographic characteristics of this population are shown in Table S2. During the follow‐up, 34 (1.8%) individuals died of SCD, 65 (3.5%) died of cardiac death, 31 (1.7%) died of non‐SCD, and 128 (6.8%) died of any cause.

Glycated hemoglobin, fasting glucose, urine albumin/creatinine ratio, QTc interval (*P*<0.001 for all), left ventricular mass index, TMV in lead I (*P*<0.01 for both), age, total cholesterol, low‐density lipoprotein, QRS duration, and TMV in lead V4 (*P*<0.05 for all) were significantly higher in the SCD group than in the SCD‐free group (Table S8). Similarly, left ventricular ejection fraction (*P*<0.001), creatinine clearance, and RR interval (*P*<0.05 for both) were significantly lower in the SCD group than in the SCD‐free group. Finally, there were significantly more individuals being given insulin (*P*<0.001), with a history of revascularization, with a Canadian Cardiovascular Society (CCS) grading of angina pectoris ≥2, with T‐wave inversions (*P*<0.01 for all), or with type 2 diabetes in the SCD group compared with the SCD‐free group (*P*<0.05; Table S8).

The C‐index of TMV in ARTEMIS was 0.635 when derived from lead I and 0.627 when derived from lead V4. When stratifying TMV in lead I according to the optimal cutoff value in ARTEMIS (TMV=2.4), positive predictive value was 3.3%, negative predictive value was 99.1%, sensitivity was 70.6%, and specificity was 61.2%. Individuals in the TMV lead I ≥2.4 group had 3.76‐fold risk (95% CI, 1.80–7.86) of dying of SCD than those in the TMV lead I <2.4 group (*P*<0.001; Figure 4B and Table 2). Finally, the optimal cutoff value for TMV in lead V4 was TMV=5, leading to a positive predictive value of 5.1%, a negative predictive value of 98.8%, a sensitivity of 47.1%, and a specificity of 83.5%. We found that individuals with TMV >5 in lead V4 had 4.42‐fold risk (95% CI, 2.25–8.67) of dying of SCD than those with TMV <5 in lead V4 (*P*<0.001; Figure 4B and Table 2).

Multivariable Cox analysis showed that left ventricular ejection fraction (*P*<0.001), CCS class ≥2 (*P*=0.029), T‐wave inversions (*P*=0.014), and TMV >5 in lead V4 (*P*=0.004) remained significantly associated with SCD in the model (Table 2). TMV >2.4 in lead I was also significant (*P*=0.032) when included in the model. The C‐index values of each model were 0.767 and 0.762, respectively (Table S9). None of the nondichotomized ECG risk markers was significantly associated with SCD (Table 2). When adjusting for non‐SCD as competing risk, we found the HRs to be similar, but now TMV per SD in lead V4 remained significantly associated with SCD in the multivariable model (Table S10). TMV did not remain significantly associated with non‐SCD, cardiac death, or all‐cause mortality, after adjusting for age, diabetes, prior revascularization, CCS class, left ventricular ejection fraction, left ventricular mass index, RR interval, QRS duration, T‐wave inversions, Tpe interval, and QTc interval (Tables S11 through S13).

When TMV in lead V4 was added to a model including age, sex, diabetes, prior revascularization, CCS class, left ventricular ejection fraction, left ventricular mass index, RR interval, QRS duration, T‐wave inversions, Tpe interval, and QTc interval, the C‐index increased from 0.743 to 0.767 (Table S9). The net reclassification improvement and integrated discrimination improvement values showed a trend toward being significant (0.284 [*P*=0.066] and 0.016 [*P*=0.060], respectively; Table S9).

## DISCUSSION

In this work, we propose, develop, and test the predictive value of the TMV index, capturing T‐wave morphologic variations with respect to a normal reference from standard resting single‐lead ECGs. We tested the association of TMV with LTVAs in a large low‐risk population from the UK Biobank, as well as with SCD in a moderate‐risk population of patients with ischemia from the ARTEMIS study. The main finding of this study is that TMV is the only measured ECG risk marker significantly associated with LTVAs in the UK Biobank, and it is a stronger SCD predictor than QTc interval and left ventricular ejection fraction in ARTEMIS when dichotomized (TMV ≥5 in lead V4, and TMV ≥2.4 in lead I).

### Clinical Translation Potential of TMV

Several T‐wave morphologic indexes have been previously proposed in the literature, like morphology complexity score,^19^ T‐wave morphologic dispersion,^20^, ^21^ the T‐wave loop dispersion,^22^ T‐wave morphologic heterogeneity,^23^ the T‐wave area dispersion,^20^, ^24^ periodic repolarization dynamics,^25^ or T‐wave morphologic restitution (TMR).^9^, ^10^, ^26^ In particular for TMR, which quantifies T‐wave morphologic changes with heart rate, in previous work we demonstrated it predicts SCD in a population of patients with chronic heart failure,^9^ and MACEs and LTVAs in the same low‐risk cohort from UK Biobank used in this study.^10^ These results, as well as those from the other described T‐wave indexes, are promising and indicate the morphology of the T‐wave has a strong LTVA prognostic value. However, any translation to large‐scale screening is limited, because this requires either the acquisition of multilead ECG (eg, T‐wave morphologic dispersion and T‐wave loop dispersion)^20^, ^21^, ^22^ or long ECG recordings with heart rate variations (eg, T‐wave alternans,^27^ periodic repolarization dynamics,^25^ the temporal variability of T‐wave morphologic heterogeneity, T‐wave morphologic dispersion, and T‐wave area dispersion,^28^ or TMR^9^, ^10^, ^26^). The objective of this work was to propose an index able to quantify T‐wave morphologic variations with respect to a normal reference from short single‐lead ECGs at rest, with a similar ease of measurement as the QRS duration or QTc or Tpe interval, to enable clinical translation and application in the community. We demonstrate the potential for clinical translation of TMV for risk stratification, and future work will compare the predictive value of TMV with the previously reported T‐wave indexes.

### TMV Predicts LTVA in a Low‐Risk Population

In the low‐risk cohort from UK Biobank, well‐established predictors of risk, like resting heart rate, QRS duration, QTc interval, T‐wave inversions, or Tpe interval, did not remain significantly associated with LTVAs, unlike TMV (Table 1). This confirms our hypothesis that the overall morphology of a single‐lead T‐wave at rest contains additional information about LTVA risk than traditional T‐wave indexes. In addition, TMV remained significantly associated with MACEs, although with a weaker HR value than that with LTVAs (Table S5), and the HR was even lower for non‐LTVA events (Table S6). This, combined with the fact that TMV did not remain significantly associated with all‐cause mortality (Table S7), suggests that TMV could better discriminate LTVA events than QTc interval, which had a similar HR across all secondary end points (Tables S5 through S7).

### TMV Predicts SCD in a Moderate‐Risk Population

In moderate‐risk patients from ARTEMIS, TMV ≥5 in lead V4 or TMV ≥2.4 in lead I was more strongly associated with SCD than known SCD risk factors, like reduced left ventricular ejection fraction or the QTc interval (Table 2). This finding would support the hypothesis that SCD manifests as a combination of mechanical and electrical abnormalities in the heart under a coronary artery disease scenario.^3^ TMV did not remain significantly associated with non‐SCD, cardiac death, or all‐cause mortality after adjusting for traditional risk factors (Tables S11 through S13). These findings would further support the ability of TMV to distinguish between SCD and non‐SCD events. Coronary artery disease is a major contributor of SCD, as well as of other cardiovascular pathologies.^13^ Therefore, the challenge in a moderate‐risk population with coronary artery disease, like the ARTEMIS study, is to identify those patients who are at higher risk of experiencing SCD.^29^ Our results in ARTEMIS show that SCD victims with diagnosed coronary artery disease had significantly larger T‐wave morphologic variations with respect to normality in resting conditions, quantified by TMV, than patients who did not experience SCD. These patients could, thus, benefit from specific preventive measures, like the implantation of cardioverters‐defibrillators.

### Electrophysiological Hypothesis Behind TMV

Previous studies have shown that the T‐wave morphology reflects dispersion of ventricular repolarization,^5^ which is an indicator of risk for life‐threatening ventricular arrhythmia. Changes in dispersion of ventricular repolarization with heart rate (ie, restitution of dispersion of ventricular repolarization) have been reported to be associated with LTVA in a higher degree.^30^ TMR quantifies T‐wave morphologic changes with heart rate following the hypothesis it would reflect the restitution of dispersion of repolarization, and we demonstrated its strong association with SCD^9^ and LTVA.^10^ However, the clinical translation of TMR is limited because ECG recordings during heart rate variations are not widely available.

TMV, instead, has been developed on the basis of the hypothesis that it reflects dispersion of ventricular repolarization at rest. In particular, we hypothesized that by comparing an average T‐wave with the corresponding sex‐, heart rate–, and lead‐specific reference T‐wave morphology, any variation attributable to sex, heart rate, or lead would be removed, and the remaining variability would be mainly attributable to dispersion of ventricular repolarization. The benefit of TMV over TMR is that, similarly as the QT or Tpe interval, it can be derived from a single‐lead, 10‐second ECG recording.

Recent electrophysiological publications have studied the mechanisms underlying the T‐wave and specific indexes,^5^, ^31^ and genome‐wide association studies have investigated the genetics and biology underlying traditional T‐wave indexes,^32^, ^33^, ^34^, ^35^ as well as TMR,^10^ uncovering important genes and pathways linking these ECG markers with risk. Future electrophysiological and genetic studies are needed to confirm the electrophysiological mechanisms underlying TMV.

### Potential for Inclusion in SCD Predictive Scores

Several prediction scores integrating ECG risk markers have been proposed,^29^, ^36^, ^37^ but these currently only include traditional ECG risk markers based on specific features of the T‐wave and, hence, ignore the important arrhythmogenic information contained in the overall morphology, as shown in this study. Early identification of individuals at risk may improve if novel indexes, such as TMV, exploit the T‐wave morphology from widely available standard ECGs. However, although the addition of TMV to a model including sex, age, type 2 diabetes, prior revascularization, CCS class, left ventricular ejection fraction, left ventricular mass index, RR interval, T‐wave inversions, QRS duration, Tpe interval, and QTc interval significantly increased the C‐index, the increment in net reclassification improvement and integrated discrimination improvement values did not reach statistical significance (Table S9).

A risk marker with strong potential for clinical translation would require an adequate specificity.^38^ In UK Biobank, we obtain sensitivity and specificity values of 44.6% and 66.5%, respectively. In studies where the number of events is low, like in UK Biobank, with only 0.4% of LTVA cases, it is frequently only possible to obtain high‐specificity values at the expense of sensitivity values in the range of 25% to 50%.^38^ If sensitivity was higher, the specificity would have to be lower, reducing the clinical utility of the marker in this population. On the contrary, in ARTEMIS, where the event rate is higher (1.8%), we observe sensitivity and specificity values of 70.6% and 61.2%, respectively.

### Strengths and Limitations

Our study has several strengths, including significant sample size in UK Biobank, rigorous adjudication of modes of death in ARTEMIS, robust and automated algorithms to derive the ECG markers, and testing in 2 different cohorts, a low‐ and a moderate‐SCD risk population. In addition, the derivation of TMV and its association with events in ARTEMIS were performed in a blinded manner. However, there are also limitations. Continuous TMV was independently predictive in UK Biobank but not in ARTEMIS, where only the dichotomized TMV was predictive in the multivariable models. The median (interquartile range) of TMV in lead V4 was 4.1 (5) in victims of SCD and 2.5 (2.2) in the SCD‐free group, as shown in Table S8. These values are 1.8 (1.3) for LTVA victims and 1.6 (1.1) for the rest in UK Biobank (Table S3). This could suggest that there is a nonlinear distribution of risk within ARTEMIS, with a cluster of individuals at risk with high values of TMV. Therefore, progressive increments of TMV might not have independent prognostic value. However, TMV significantly predicted SCD in ARTEMIS when competing risks were considered (Table S10). In addition, the optimal cutoff values were different across both UK Biobank and ARTEMIS. This reflects different characteristics of the 2 populations: individuals in UK Biobank do not have underlying cardiovascular disease, whereas patients in ARTEMIS have coronary artery disease, and many of them had a documented prior myocardial infarction, mostly non–Q‐wave infarctions. This may have induced dynamic repolarization changes indirectly, captured by TMV, that are not present in UK Biobank. Moreover, we used hospital episode statistics to define the outcomes in UK Biobank, so we cannot rule out the possibility that some participants included in the LTVA group may have experienced nonarrhythmic events. However, hospital episode statistics are the most reliable option in large studies, and we would expect any potential misclassification to be nondifferential and thus bias our results conservatively toward the null. Besides, our results from the competing risk regression analyses support the reliability of the association with LTVA in UK Biobank. Also, the risk factors included as covariates in the survival analyses models differed across UK Biobank and ARTEMIS analyses (as they were different cohort studies). Only 1 lead was available from the UK Biobank cohort, so we were not able to test the risk stratification value of TMV in other leads; future studies will evaluate the impact of the selected lead on TMV and its predictive value. Finally, given the relatively homogeneous ethnic background of patients in the UK Biobank and ARTEMIS cohorts, our findings warrant evaluation in cohorts with greater diversity.

## CONCLUSIONS

In conclusion, TMV, an ECG index quantifying T‐wave morphologic variations with respect to a normal reference from a single beat from a single‐lead ECG, is significantly associated with LTVAs in a large low‐risk population, and is a stronger SCD predictor than traditional risk factors in a moderate‐risk population when dichotomized. We demonstrate the potential clinical translation of TMV for risk stratification in large‐scale screening studies.

## Sources of Funding

Dr Ramírez acknowledges the “María Zambrano” fellowship support from the European Union–NextGenerationEU and the support from the Marie Sklodowska‐Curie grant 786833. We also wish to acknowledge support by the Medical Research Council grant MR/N025083/1. Dr Lambiase is supported by University College London/University College London Hospital Biomedicine National Institute of Health Research (NIHR). Drs Tinker and Munroe acknowledge the NIHR Cardiovascular Biomedical Research Centre at Barts and Queen Mary University of London, UK. Dr Junttila acknowledges funding from Academy of Finland, Sigrid Juselius Foundation, and Finnish Foundation for Cardiovascular Research.

## Disclosures

None.

## Supporting information

Data S1
Tables S1–S13
Figures S1–S2
References 39, 40, 41
