# Supplementary material for: ECG T‐Wave Morphologic Variations Predict Ventricular Arrhythmic Risk in Low‐ and Moderate‐Risk Populations
Source: J Am Heart Assoc. 2022 Aug 29;11(17):e025897. doi: 10.1161/JAHA.121.025897 (PMC9496440; doi:10.1161/JAHA.121.025897)

SUPPLEMENTAL MATERIAL

Data S1.

Supplemental Methods

Reference cohort, UK Biobank

UK Biobank is a prospective study of 488,377 individuals, comprising relatively even numbers of men and women aged 40 to 69 years old at recruitment (2006–2008). The UK Biobank study has approval from the North West Multi-Centre Research Ethics Committee, and all participants provided informed consent(39). The work was undertaken as part of UK Biobank application 8256.

Ten second 12-lead electrocardiogram (ECG) recordings at rest were acquired from a sub-cohort of 36,507 individuals in the UK Biobank (middle-aged UK volunteers) who participated in an imaging study (05/2014 – 03/2019; the collection is ongoing). Individuals were excluded if they were admitted to hospital due to any of the International Classification of Diseases, Tenth Revision (ICD-10) or if they had an intervention matching any of the Office of Population Censuses and Surveys Classification of Interventions and Procedures version 4 (OPCS-4) codes in Table S1, or a poor ECG quality, leading to a total of 23,962 participants remaining in the reference cohort (Figure 2).

Low-risk test cohort, UK Biobank

An independent cohort of 95,216 individuals in the UK Biobank were invited for an exercise stress test, including 15 s of resting ECG acquired with a 1-lead (lead I, 2009) ECG device. Complete ECG recordings from 58,839 individuals were available (Figure 2). Similarly, as for the reference cohort, individuals were excluded if they had experienced a previous cardiovascular event (matching the codes from Table S1), or if the ECG had poor quality, leading to 51,794 individuals included in the analyses.

The primary endpoint was life-threatening ventricular arrhythmias (LTVAs), defined as ventricular arrhythmic (VA) mortality or admission to hospital with a LTVA diagnosis. ICD-10 and OPCS-4 codes used to define LTVA are presented in Table S1. The secondary endpoints

were major adverse cardiovascular events (MACE, including mortality or admissions to hospital), including all ICD-10 or OPCS-4 codes in Table S1, non-LTVA cardiac death and all-cause mortality. Follow-up was from the study inclusion date until June 22, 2020.

Moderate-risk test cohort, ARTEMIS

The ARTEMIS database consists of 1,946 patients from Finland with coronary artery disease(13). Examinations during the enrolment visit included 12-lead ECGs acquired during an exercise stress test (also with 15 s at rest) for 1,886 participants (Figure 2, only leads I and V4 were analysed in this work). Fifty-one subjects were excluded because of no ECG at rest or poor ECG quality, leading to 1,835 individuals included in the analyses (Figure 2). All enrolled patients gave informed consent, and the institutional ethics committee approved the study. The study complies with the Declaration of Helsinki.

The primary endpoint was sudden cardiac death (SCD) or resuscitation from sudden cardiac arrest, whichever occurred first. The definition for SCD was a witnessed death within 1 hour of the onset of symptoms. For unwitnessed deaths, the definition was last being seen alive and stable 24 hours before discovery. The secondary endpoints were cardiac death, including SCD, aborted sudden cardiac arrest, and non-SCD, whichever occurred first, and all-cause mortality. Follow-up was 5 years(13).

ECG pre-processing

Pre-processing of the ECG signals included low-pass filtering at 50 Hz to remove electric and muscle noise but still allow QRS detection(40). Baseline wander was removed by further high-pass filtering of the ECG signals at 0.5 Hz. We then signal-averaged the heartbeats within a window of 15 s at rest to attenuate noise and artefacts and reveal small variations in the QRS-T-waveform. The onset, peak, and end timings of the waveforms were located using the same bespoke software as in previous studies(31, 41).

Deriving normal T-wave morphology references

Initially, the reference cohort was divided into females and males. Then, we further clustered the individuals within each sex group by their average RR interval (inverse of heart rate, Figure 2). For each individual within each cluster, the T-wave (from its onset to its end) was further low-pass filtered at 20 Hz to remove remaining out-of-band high frequency components that could potentially corrupt its morphology. Finally, we derived sex-, heart rate- and lead-specific T-wave references by averaging all T-waves within each cluster using a warping-based methodology(14).

TMV index, T-wave morphology variations with respect to a normal reference

For each participant in the low- and moderate-risk cohorts (UK Biobank and ARTEMIS, respectively), we compared their average T-wave with their corresponding sex-, RR- and lead-specific (lead I in UK Biobank, and leads I and V4 in ARTEMIS) normal T-wave morphology reference using dynamic programming to find the warping function that optimally aligns both T-wave morphologies(14) (Figure 3). For each individual, we derived the TMV index, quantifying T-wave morphology variations with respect to a normal reference (Figure 3). The specific equation of TMV is as follows:

$$TMV = \frac{1}{N_r}\sum_{n=1}^{N_r}\left|\gamma^*(t^r(n)).\frac{f^r(t^r(n))}{\max(f^r(t^r(n)))} - t^r(n)\right|$$

, where $\gamma^*(t^r)$ is the optimal warping function relating the average T-wave from each participant to its corresponding sex- and RR- normal T-wave morphology reference ($f^r(t^r)$, of length N_r), with an additional weighted that has recently proved to be more robust against noise(15).

We, then, followed the same procedure to derive TMV in the moderate-risk cohort (ARTEMIS) from lead I (to ease comparisons across cohorts) and from lead V4 (optimal to capture ventricular repolarization as it usually shows the T-wave with the highest energy, but not available in UK Biobank). The derivation of TMV and its association with events in ARTEMIS was performed in a blindly manner.

Statistical Analyses

In UK Biobank, the QT and Tpe intervals were measured as the intervals between the QRS-onset and the T-wave end, and between the T-wave peak and the T-wave end, respectively, from the averaged heartbeat at rest. Then, we corrected the QT interval using Bazett formula(16). We additionally derived the marker T-wave inversion, which indicated a change in the polarity of the T-waves(6), and the QRS duration. In ARTEMIS, these ECG indices were automatically derived using custom made software(17). Missing data were imputed using the "mice" package in R, provided a missing rate < 10%. Variables with a higher rate of missingness were excluded.

The 2-tailed Mann-Whitney and Fisher exact tests were used for Univariable comparison of quantitative and categorical data, respectively. The C-index was calculated to estimate the performance of TMV in both UK Biobank and ARTEMIS. We estimated the optimal cut-off values for TMV in both low- and moderate-risk cohorts based on the highest sum of specificity and sensitivity above median values with at least 20% sensitivity, as in previous studies(10). For these optimal cut-off values, we provide values of positive predictive value (PPV), negative predictive value (NPV), sensitivity and specificity. Kaplan-Meier curves were derived using the optimal cut-off values, with a comparison of cumulative events performed by using log-rank tests, and plotted using the "survminer" package in R.

Univariable and multivariable Cox regression analyses were performed to determine the predictive value of the risk markers. Models were adjusted by risk factors shown in Table 1 (UK Biobank) and Table 2 (ARTEMIS). All continuous variables were standardized to a mean of 0 and standard deviation (SD) of 1 to allow for comparisons in the Cox models. Only the variables with a significant association with the endpoint in Univariable analysis were included in the multivariable model. Stepwise regression analysis was then performed to only retain the variables independently associated with the outcome. Individuals who died from causes not included in the primary end point were censored at the time of death. In ARTEMIS, TMV

measured from leads I or V4 were entered one at a time into the multivariable model. Competing risks survival analyses (Gray's method)(18) were also conducted using approaches of LTVA versus a non-LTVA event in UK Biobank and SCD vs. death from a cause other than SCD (non-SCD) in ARTEMIS. The C-index, as well as the net reclassification improvement (NRI) index and the integrated discrimination improvement (IDI) index were calculated to estimate the improvement of adding the strongest TMV index (measured on lead I or on lead V4). A value of $P < 0.05$ was considered statistically significant. Statistical analyses were performed using R version 4.0.2.

Table S1: Codes used to define the MACE and LTVA groups

Myocardial Infarction		
	ICD10 codes	Definition
Myocardial Infarction	I21	Acute myocardial infarction
	I21.0	Acute transmural myocardial infarction of anterior wall
	I21.1	Acute transmural myocardial infarction of inferior wall
	I21.2	Acute transmural myocardial infarction of other sites
	I21.3	Acute transmural myocardial infarction of unspecified site
	I21.4	Acute subendocardial myocardial infarction
	I21.9	Acute myocardial infarction, unspecified
	I22	Subsequent myocardial infarction
	I22.0	Subsequent myocardial infarction of anterior wall
	I22.1	Subsequent myocardial infarction of inferior wall
	I22.8	Subsequent myocardial infarction of other sites
	I22.9	Subsequent myocardial infarction of unspecified site
	I23	Certain current complications following acute myocardial infarction
	I23.0	Haemopericardium as current complication following acute myocardial infarction
	I23.1	Atrial septal defect as current complication following acute myocardial infarction
	I23.2	Ventricular septal defect as current complication following acute myocardial infarction
	I23.3	Rupture of cardiac wall without haemopericardium as current complication following acute myocardial infarction

I23.4	Rupture of chordae tendineae as current complication following acute myocardial infarction
I23.5	Rupture of papillary muscle as current complication following acute myocardial infarction
I23.6	Thrombosis of atrium , auricular appendage and ventricle as current complications following acute myocardial infarction
I23.8	Other current complications following acute myocardial infarction
**ICD9 codes**	**Definition**
4109	Acute myocardial infarction
**Operation (self-reported)**	**Definition**
1070	Coronary angioplasty (ptca) + stent
1095	Coronary artery bypass grafts (cabg)
1523	Triple Heart bypass
**OPCS4**	**Definition**
K40	Saphenous vein graft replacement of coronary artery
K40.1	Saphenous vein graft replacement of one coronary artery
K40.2	Saphenous vein graft replacement of two coronary arteries
K40.3	Saphenous vein graft replacement of three coronary arteries
K40.4	Saphenous vein graft replacement of four or more coronary arteries
K40.9	Unspecified saphenous vein graft replacement of coronary artery
K41	Other autograft replacement of coronary artery
K41.1	Autograft replacement of one coronary artery NEC
K41.2	Autograft replacement of two coronary arteries NEC
K41.3	Autograft replacement of three coronary arteries NEC
K41.4	Autograft replacement of four or more coronary arteries NEC
K42	Allograft replacement of coronary artery

K42.4	Allograft replacement of four or more coronary arteries
K44	Other replacement of coronary artery
K44.1	Replacement of coronary arteries using multiple methods
K44.2	Revision of replacement of coronary artery
K44.9	Unspecified other replacement of coronary artery
K45	Connection of thoracic artery to coronary artery
K45.1	Double anastomosis of mammary arteries to coronary arteries
K45.2	Double anastomosis of thoracic arteries to coronary arteries NEC
K45.3	Anastomosis of mammary artery to left anterior descending coronary artery
K45.4	Anastomosis of mammary artery to coronary artery NEC
K45.5	Anastomosis of thoracic artery to coronary artery NEC
K45.6	Revision of connection of thoracic artery to coronary artery
K45.8	Other specified connection of thoracic artery to coronary artery
K45.9	Unspecified connection of thoracic artery to coronary artery
K49	Transluminal balloon angioplasty of coronary artery
K49.1	Percutaneous transluminal balloon angioplasty of one coronary artery
K49.2	Percutaneous transluminal balloon angioplasty of multiple coronary arteries
K49.3	Percutaneous transluminal balloon angioplasty of bypass graft of coronary artery
K49.4	Percutaneous transluminal cutting balloon angioplasty of coronary artery
K49.8	Other specified transluminal balloon angioplasty of coronary artery
K49.9	Unspecified transluminal balloon angioplasty of coronary artery
K50	Other therapeutic transluminal operations on coronary artery
K50.1	Percutaenous transluminal laser coronary angioplasty

	K50.2	Percutaneous transluminal coronary thrombolysis using streptokinase
	K50.3	Percutaneous transluminal injection of therapeutic substance into coronary artery NEC
	K50.4	Percutaenous transluminal atherectomy of coronary artery
	K50.8	Other specified other therapeutic transluminal operations on coronary artery
	K50.9	Unspecified other therapeutic transluminal operations on coronary artery
	K75	Percutaneous transluminal balloon angioplasty and insertion of stent into coronary artery
	K75.1	Percutaneous transluminal balloon angioplasty and insertion of 1-2 drug-eluting stents into coronary artery
	K75.2	Percutaneous transluminal balloon angioplasty and insertion of 3 or more drug-eluting stents into coronary artery
	K75.3	Percutaneous transluminal balloon angioplasty and insertion of 1-2 stents into coronary artery
	K75.4	Percutaneous transluminal balloon angioplasty and insertion of 3 or more stents into coronary artery NEC
	K75.8	Other specified percutaenous transluminal balloon angioplasty and insertion of stent into coronary artery
	K75.9	Unspecified percutaenous transluminal balloon angioplasty and insetion of stent into coronary artery
**Heart Failure**		
**Heart Failure**	**ICD10 codes**	**Definition**
	I13.0	Hypertensive heart and renal disease with both (congestive) heart failure

	I13.2	Hypertensive heart and renal disease with both (congestive) heart failure and renal failure
	I50	Heart failure
	I50.0	Congestive heart failure
	I50.1	Left ventricular failure
	I50.9	Heart failure, unspecified
	**ICD9 codes**	**Definition**
	4280	Congestive heart failure
	4281	Left heart failure
	4289	Heart failure, unspecified
	**OPCS4**	**Definition**
	K59.6	Implantation of cardioverter defibrillator using three electrode leads
	K61.7	Implantation of biventricular cardiac pacemaker system
	K60.7	Implantation of intravenous biventricular cardiac pacemaker system
**Ventricular Arrhythmia**		**Life Threatening Ventricular Arrhythmia**
	**ICD10 codes**	**Definition**
	I47.2	Ventricular tachycardia
	I49.0	Ventricular fibrillation and flutter
	I46.0	Cardiac arrest with successful resuscitation
	I46.1	Sudden cardiac death, so described
	I46.9	Cardiac arrest, unspecified
	I47.0	Re-entry ventricular arrhythmia
	**OPCS4**	**Definition**
	K57.6	Percutaneous transluminal ablation of ventricular wall
	K64.1	Percutaneous radiofrequency ablation of epicardium
	X50.3	Advanced cardiac pulmonary resuscitation
	X50.4	Evaluation of cardioverter defibrillator

ICD Implant		
	**OPCS4**	**Definition**
ICD Implant	K59	Cardioverter defibrillator introduced through vein
	K59.1	Implantation of cardioverter defibrillator using one electrode lead
	K59.2	Implantation of cardioverter defibrillator using two electrode leads
	K59.3	Resiting of lead of cardioverter defibrillator
	K59.4	Renewal of cardioverter defibrillator
	K59.6	Implantation of cardioverter defibrillator using three electrode leads
	K59.8	Other specified cardioverter defibrillator introduced through the vein
	K59.9	Unspecified cardioverter defibrillator introduced through the vein
	K72	Other cardioverter defibrillator
	K72.1	Implantation of subcutaenous cardioverter defibrillator
	K72.3	Renewal of subcutaneous cardioverter defibrillator

Table S2: Patient characteristics in the UK Biobank and ARTEMIS cohorts

	UK Biobank cohort	ARTEMIS cohort
**Study characteristics**		
Number of subjects, N	51,794	1,835
Median follow-up (IQR), months	121.9 (3.7)	60 (0)
Ventricular arrhythmic events, n(%)/SCD, n(%)	220 (0.4)	34 (1.8)
MACE, n(%)/CD, n(%)	1,591 (3.1)	65 (3.5)
non-ventricular arrhythmic events, n(%)/non-SCD, n(%)	1,371 (2.6)	31 (1.7)
All-cause mortality events, n(%)	1,547 (3.0)	128 (6.8)
**Subject characteristics**		
Median age (IQR), years	58 (13)	67 (12)
Males, n(%)	23,954 (46.2)	1,257 (67.1)
Diabetes mellitus, n(%)	2,006 (3.9)	775 (41.4)
Median BMI (IQR), kg/m2	26.4 (5.2)	28 (6)
Median systolic blood pressure (IQR), mmHg	135.5 (24)	146 (33)
Median diastolic blood pressure (IQR), mmHg	81.5 (13)	80 (15)
Previous or current smoker, n(%)	22,040 (42.6)	944 (50.4)
History of prior myocardial infarction, n(%)	0 (0)	877 (46.8)
History of revascularization, n(%)	0 (0)	1,465 (78.3)
CCD class ≥ 2, n(%)	0 (0)	777 (41.5)
Median Syntax Score (IQR)	0 (0)	0 (5)
Median left ventricular ejection fraction (IQR), %	Not available	65.6 (10.2)
Median left ventricular mass index (IQR), g/m2	Not available	104.2 (33.5)
Beta blockers, n(%)	0 (0)	1,611 (86.1)

angiotensin converting enzyme inhibitors or receptor blockers, n(%)	0 (0)	1,250 (66.8)
Calcium channel blockers, n(%)	0 (0)	446 (23.8)
Diuretics, n(%)	0 (0)	607 (32.4)
Statins, n(%)	0 (0)	1,680 (89.7)
Insulin, n(%)	0 (0)	203 (10.8)
Median glycated hemoglobin (IQR), mmol/mol	35 (4.9)	43.2 (9.8)
Median fasting glucose (IQR), mmol/L	4.978 (0.605)	5.9 (1.5)
Median total cholesterol (IQR), mmol/L	5.717 (1.452)	3.8 (1)
Median low-density lipoprotein cholesterol (IQR), mmol/L	3.546 (1.127)	2.1 (0.8)
Median high-density lipoprotein cholesterol (IQR), mmol/L	1.459 (0.511)	1.22 (0.41)
Median Triglycerides (IQR), mmol/L	1.414 (0.998)	1.21 (0.76)
Median creatinine clearance (IQR), mL/min	71.30 (19.40)	87.9 (41.4)
Median Urine-Albumin/Creatinine-ratio (IQR)	0.642 (0.172)	0.9 (0.8)

BMI, body mass index; CCD, Canadian Cardiovascular Society grading of angina pectoris; CD, cardiac death; IQR, interquartile range; SCD, sudden cardiac death.

Table S3: Characteristics of the study population in the LTVA and in the non-LTVA groups in UK Biobank

	**LTVA**	**Non-LTVA**	
**Characteristics**	N = 220	N = 51,574	**P-value**
Median age (IQR), years	63 (8)	58 (13)	**<0.001**
Males, n(%)	159 (72.3)	23,795 (46.1)	**<0.001**
Diabetes mellitus, n(%)	9 (4.1)	2,011 (3.9)	0.861
Median BMI (IQR), kg/m2	27.1 (5.0)	26.4 (5.3)	**0.019**
Median systolic blood pressure (IQR), mmHg	143.0 (20.1)	135.0 (24.0)	**<0.001**
Median diastolic blood pressure (IQR), mmHg	83.0 (12.0)	81.5 (12.5)	**0.018**
Previous or current smoker, n(%)	102 (46.4)	21,940 (42.5)	0.274
Median glycated hemoglobin (IQR), mmol/mol	36.1 (5.9)	35.0 (4.9)	**0.005**
Median fasting glucose (IQR), mmol/L	5.020 (0.620)	4.979 (0.606)	0.486
Median total cholesterol (IQR), mmol/L	5.637 (1.316)	5.721 (1.461)	0.499
Median low-density lipoprotein cholesterol (IQR), mmol/L	3.519 (1.112)	3.548 (1.129)	0.980
Median high-density lipoprotein cholesterol (IQR), mmol/L	1.367 (0.478)	1.460 (0.512)	**<0.001**
Median triglycerides (IQR), mmol/L	1.544 (1.054)	1.410 (0.995)	**0.010**
Median creatinine clearance (IQR), mL/min	78.70 (19.98)	71.30 (19.40)	**<0.001**
Median U-Albumin/Creatinine-ratio (IQR), d.u.	0.581 (0.166)	0.642 (0.172)	**<0.001**
Median Resting RR interval (IQR), s	0.848 (0.186)	0.861 (0.174)	0.107
Median QRS duration (IQR), s	0.096 (0.021)	0.092 (0.022)	0.150
T-wave inversions, n(%)	1 (0.5)	15 (0.0)	0.066
Median resting Tpe interval (IQR), s	0.063 (0.014)	0.062 (0.012)	0.715
Median resting QTc interval (IQR), s	0.395 (0.030)	0.395 (0.030)	0.209
Median TMV (IQR), s	1.843 (1.315)	1.642 (1.107)	**0.003**

IQR, interquartile range; BMI, body mass index; LTVA, life-threatening ventricular arrhythmia; QTC, corrected QT interval; Tpe, T-peak-to-T-end interval; TMV, T-wave morphology variations with respect to a normal reference.

Significant differences are indicated in bold.

Table S4: LTVA versus non-LTVA competing risk regression in UK Biobank

	Univariable	Multivariable
**Univariate**	**Hazard ratio (95%CI)**	**Hazard ratio (95%CI)**
TMV (per SD)	1.19 (1.09-1.29), p<0.001	1.13 (1.04-1.23), p=0.003

CI = confidence interval. Adjusted for sex, age, systolic blood pressure and creatinine (the significant variables in the model in Table 1).

Table S5: Association with major adverse cardiovascular events in UK Biobank

	UK Biobank			
	Univariate		Multivariate	
Trait	Hazard ratio (95% CI)	*P* value	Hazard ratio (95% CI)	P value
**Sex**	**3.065 (2.747 - 3.419)**	**<0.001**	**2.383 (2.093 - 2.714)**	**<0.001**
**Age (per 1 SD)**	**1.929 (1.818 - 2.047)**	**<0.001**	**1.729 (1.624 - 1.842)**	**<0.001**
**Diabetes mellitus (yes)**	**2.646 (2.236 - 3.130)**	**<0.001**	**1.650 (1.328 - 2.050)**	**<0.001**
**BMI (per 1 SD)**	**1.281 (1.228 - 1.337)**	**<0.001**	**1.106 (1.048 - 1.168)**	**<0.001**
**SBP (per 1 SD)**	**1.481 (1.412 - 1.552)**	**<0.001**	**1.163 (1.103 - 1.227)**	**<0.001**
DBP (per 1 SD)	1.253 (1.194 - 1.315)	<0.001	-	-
**Previous or current smoker (yes)**	**1.424 (1.291 - 1.571)**	**<0.001**	**1.147 (1.038 - 1.267)**	**0.007**
**Glycated hemoglobin (per 1 SD)**	**1.209 (1.177 - 1.242)**	**<0.001**	**1.115 (1.057 - 1.176)**	**<0.001**
**Glucose (per 1 SD)**	**1.096 (1.061 - 1.131)**	**<0.001**	**0.920 (0.873 - 0.969)**	**0.002**
Cholesterol (per 1 SD)	1.006 (0.958 - 1.057)	0.805	-	-
**LDL (per 1 SD)**	**1.089 (1.037 - 1.143)**	**<0.001**	**1.201 (1.143 - 1.262)**	**<0.001**
**HDL (per 1 SD)**	**0.654 (0.618 - 0.693)**	**<0.001**	**0.822 (0.771 - 0.877)**	**<0.001**
Triglycerides (per 1 SD)	1.269 (1.224 - 1.315)	<0.001	-	-
**Creatinine (per 1 SD)**	**1.137 (1.122 - 1.153)**	**<0.001**	**1.090 (1.050 - 1.131)**	**<0.001**
**Albumina (per 1 SD)**	**0.938 (0.893 - 0.986)**	**0.011**	**0.932 (0.885 - 0.981)**	**0.007**
Alb/Creatinine ratio (per 1 SD)	0.650 (0.616 - 0.686)	<0.001	-	-
Resting RR interval (per 1 SD)	0.964 (0.917 - 1.013)	0.148	-	-
QRS duration (per 1 SD)	1.101 (1.048 - 1.156)	<0.001	-	-
**T-wave inversion (yes)**	**11.919 (4.954 - 28.675)**	**<0.001**	**6.039 (2.329 - 15.659)**	**<0.001**
Resting Tpe interval (per 1 SD)	1.033 (0.984 - 1.085)	0.189	-	-
**Resting QTc interval (per 1 SD)**	**1.128 (1.076 - 1.183)**	**<0.001**	**1.110 (1.054 - 1.168)**	**<0.001**
**TMV (per 1 SD, lead I)**	**1.174 (1.131 - 1.218)**	**<0.001**	**1.055 (1.014 - 1.097)**	**0.007**

BMI, body mass index; CI, confidence interval; HTN, hypertension; QTc, corrected QT, using Bazett's formula; SD, standard deviation; TMV, T-wave variations with respect to a normal reference; Tpe, T-peak-to-T-end. Significant variables in the Multivariable model are indicated in bold.

Table S6: Association with non-LTVA in UK Biobank

Trait	Univariate		Multivariate	
	Hazard ratio (95% CI)	*P* value	Hazard ratio (95% CI)	P value
**Sex**	**3.079 (2.737 - 3.464)**	**<0.001**	**2.371 (2.059 - 2.731)**	**<0.001**
**Age (per 1 SD)**	**1.934 (1.814 - 2.061)**	**<0.001**	**1.737 (1.623 - 1.859)**	**<0.001**
**Diabetes mellitus (yes)**	**2.922 (2.455 - 3.479)**	**<0.001**	**1.821 (1.450 - 2.287)**	**<0.001**
**BMI (per 1 SD)**	**1.304 (1.246 - 1.364)**	**<0.001**	**1.119 (1.056 - 1.186)**	**<0.001**
**SBP (per 1 SD)**	**1.484 (1.410 - 1.561)**	**<0.001**	**1.161 (1.096 - 1.230)**	**<0.001**
DBP (per 1 SD)	1.275 (1.210 - 1.343)	**<0.001**	-	-
**Previous or current smoker (yes)**	**1.474 (1.326 - 1.639)**	**<0.001**	**1.183 (1.063 - 1.318)**	**0.002**
**Glycated hemoglobin (per 1 SD)**	**1.219 (1.185 - 1.253)**	**<0.001**	**1.116 (1.055 - 1.181)**	**<0.001**
**Glucose (per 1 SD)**	**1.102 (1.066 - 1.139)**	**<0.001**	**0.918 (0.869 - 0.970)**	**0.002**
Cholesterol (per 1 SD)	1.013 (0.961 - 1.068)	0.632	-	-
LDL (per 1 SD)	1.102 (1.046 - 1.161)	**<0.001**	**1.226 (1.162 - 1.292)**	**<0.001**
**HDL (per 1 SD)**	**0.635 (0.597 - 0.676)**	**<0.001**	**0.799 (0.745 - 0.858)**	**<0.001**
Triglycerides (per 1 SD)	1.285 (1.237 - 1.335)	<0.001	-	-
**Creatinine (per 1 SD)**	**1.136 (1.119 - 1.153)**	**<0.001**	**1.079 (1.033 - 1.128)**	**<0.001**
Albumina (per 1 SD)	**0.943 (0.894 - 0.994)**	**0.031**	**0.937 (0.887 - 0.990)**	**0.021**
Alb/Creatinine ratio (per 1 SD)	0.655 (0.618 - 0.694)	<0.001	-	-
Resting RR interval (per 1 SD)	0.975 (0.925 - 1.029)	0.364	-	-
QRS duration (per 1 SD)	1.100 (1.043 - 1.160)	<0.001		
T-wave inversion (yes)	10.871 (4.074 - 29.006)	**<0.001**	**5.705 (1.979 - 16.444)**	**0.001**
Resting Tpe interval (per 1 SD)	1.040 (0.987 - 1.095)	0.145	-	-
**Resting QTc interval (per 1 SD)**	**1.135 (1.079 - 1.194)**	**<0.001**	**1.114 (1.054 - 1.177)**	**<0.001**
**TMV (per 1 SD, lead I)**	**1.172 (1.126 - 1.220)**	**<0.001**	**1.050 (1.007 - 1.095)**	**0.024**

BMI, body mass index; CI, confidence interval; HTN, hypertension; QTc, corrected QT, using Bazett's formula; SD, standard deviation; TMV, T-wave variations with respect to a normal reference; Tpe, T-peak-to-T-end.

Significant variables in the Multivariable model are indicated in bold.

Table S7: Association with all-cause mortality in UK Biobank

Trait	Univariate		Multivariate	
	Hazard ratio (95% CI)	*P* value	Hazard ratio (95% CI)	P value
**Sex**	**1.596 (1.443 - 1.766)**	**<0.001**	**1.421 (1.271 - 1.589)**	**<0.001**
**Age (per 1 SD)**	**2.343 (2.196 - 2.501)**	**<0.001**	**2.154 (2.012 - 2.306)**	**<0.001**
**Diabetes mellitus (yes)**	**2.177 (1.812 - 2.616)**	**<0.001**	**1.410 (1.160 - 1.713)**	**<0.001**
BMI (per 1 SD)	1.104 (1.053 - 1.158)	<0.001		
**SBP (per 1 SD)**	**1.398 (1.333 - 1.467)**	**<0.001**	**1.086 (1.030 - 1.145)**	**<0.001**
DBP (per 1 SD)	1.144 (1.089 - 1.202)	<0.001		
**Previous or current smoker (yes)**	**1.599 (1.447 - 1.767)**	**<0.001**	**1.351 (1.221 - 1.495)**	**<0.001**
Glycated hemoglobin (per 1 SD)	1.171 (1.135 - 1.208)	<0.001		
Glucose (per 1 SD)	1.110 (1.078 - 1.144)	<0.001		
**Cholesterol (per 1 SD)**	**0.906 (0.862 - 0.954)**	**<0.001**	**0.934 (0.885 - 0.986)**	**0.013**
LDL (per 1 SD)	0.913 (0.868 - 0.961)	<0.001		
HDL (per 1 SD)	0.890 (0.845 - 0.938)	<0.001		
**Triglycerides (per 1 SD)**	**1.126 (1.078 - 1.175)**	**<0.001**	**1.060 (1.009 - 1.113)**	**0.022**
Creatinine (per 1 SD)	1.093 (1.064 - 1.123)	<0.001		
**Albumina (per 1 SD)**	**0.869 (0.826 - 0.913)**	**<0.001**	**0.900 (0.855 - 0.947)**	**<0.001**
Alb/Creatinine ratio (per 1 SD)	0.843 (0.801 - 0.888)	<0.001		
**Resting RR interval (per 1 SD)**	**0.909 (0.863 - 0.957)**	**<0.001**	**0.926 (0.875 - 0.980)**	**0.008**
**QRS duration (per 1 SD)**	**1.118 (1.063 - 1.175)**	**<0.001**	**1.081 (1.027 - 1.138)**	**0.003**
T-wave inversion (yes)	2.150 (0.303 - 15.271)	0.444		
Resting Tpe interval (per 1 SD)	1.013 (0.964 - 1.064)	0.617		
**Resting QTc interval (per 1 SD)**	**1.186 (1.132 - 1.242)**	**<0.001**	**1.090 (1.031 - 1.153)**	**0.002**
TMV (per 1 SD, lead I)	1.110 (1.064 - 1.158)	<0.001		

BMI, body mass index; CI, confidence interval; HTN, hypertension; QTc, corrected QT, using Bazett's formula; SD, standard deviation; TMV, T-wave variations from a normal reference; Tpe, T-peak-to-T-end.

Significant variables in the Multivariable model are indicated in bold.

Table S8: Characteristics of the study population in the SCD and in the SCD-free groups in ARTEMIS

**Characteristics**	**SCD group** N = 34	**SCD-free group** N = 1,801	**P-value**
Median age (IQR), years	70 (7)	67 (9)	**<0.05**
Males, n(%)	26 (77)	1,231 (68)	N.S.
Diabetes mellitus, n(%)	21 (62)	754 (42)	**<0.05**
Median BMI (IQR), kg/m2	27 (3)	28 (5)	N.S.
Median SBP (IQR), mmHg	143 (22)	147 (25)	N.S.
Median DBP (IQR), mmHg	79 (11)	81 (12)	N.S.
Previous or current smoker, n(%)	23 (68)	921 (51)	N.S.
History of prior myocardial infarction, n(%)	21 (61)	856 (48)	N.S.
History of revascularization, n(%)	30 (88)	1435 (80)	**<0.01**
CCS class ≥ 2, n(%)	23 (68)	754 (42)	**<0.01**
Syntax Score	2 (0-7)	0 (0-5)	N.S.
Left ventricular ejection fraction (%)	56 (15)	64 (9)	**<0.001**
Left ventricular mass index (g/m2)	122 (27)	107 (27)	**<0.01**
Beta-blockers, n(%)	29 (85)	1,582 (88)	N.S.
Angiotensin converting enzyme inhibitors or receptor blockers, n(%)	26 (77)	1,24 (68)	N.S.
Calcium channel blockers, n(%)	10 (29)	436 (24)	N.S.
Diuretics, n(%)	16 (47)	591 (33)	N.S.
Statins, n(%)	29 (85)	1,651 (92)	N.S.

Insulin, n(%)	11 (32)	192 (11)	**<0.001**
Glycated hemoglobin (mmol/mol)	53 (22)	46 (11)	**<0.001**
Glycated hemoglobin (%)	7.0 (2.0)	6.3 (1)	**<0.001**
Fasting glucose (mmol/L)	7.5 (3.9)	6.4 (1.6)	**<0.001**
Total cholesterol (mmol/L)	4.3 (1.1)	4 (0.9)	**<0.05**
High-density lipoprotein cholesterol (mmol/L)	1.2 (0.3)	1.3 (0.3)	N.S.
Low-density lipoprotein cholesterol (mmol/L)	2.6 (1.1)	2.3 (0.8)	**<0.05**
Triglycerides (mmol/L)	1.4 (1)	1.2 (0.8)	N.S.
Creatinine clearance (mL/min)	80 (29)	94 (34)	**<0.05**
U-Albumin/Creatinine-ratio	1.6 (1.6)	0.8 (0.7)	**<0.001**
Median RR interval (IQR), s	0.950 (0.186)	1.004 (0.147)	**<0.05**
Median QRS interval (IQR), s	0.106 (0.033)	0.100 (0.016)	**<0.05**
Median QTc (IQR), s	0.441 (0.035)	0.424 (0.026)	**<0.001**
Median Tpe interval (IQR), s	0.087 (0.011)	0.088 (0.014)	N.S.
T-wave inversions, n(%)	23 (68%)	604 (34)	**<0.01**
Median TMV in Lead I (IQR), s.	2.7 (2.2)	2 (1.9)	**<0.01**
Median TMV in Lead V4 (IQR), s	4.1 (5)	2.5 (2,2)	**<0.05**

BMI, body mass index; CCS, Canadian Cardiovascular Society grading of angina pectoris; DBP, diastolic blood pressure; IQR, interquartile range; QTc, corrected QT interval; SBP,

systolic blood pressure; SCD, sudden cardiac death; Tpe, T-peak-to-T-end; TMV, T-wave morphology variations.

Significant differences are indicated in bold.

Table S9: C-index, net reclassification index (continuous) and integrated discrimination index, SCD as endpoint.

	**C-index (95%CI)**	**NRI (95%CI)**	**IDI (95%CI)**
	**HR (95%CI)**	**HR (95%CI)**	**HR (95%CI)**
Established model	0.743 (0.641-0.845)	-	-
TMV Lead I	0.743 (0.641-0.845)	0.069 (-0.135-0.263) p=0.605	0.000 (-0.001-0.007) p=0.545
TMV Lead V4	0.747 (0.643-0.851)	0.179 (-0.150-0.360) p=0.219	0.004 (-0.002-0.016) p=0.173
TMV Lead I ≥ 2.4	0.762 (0.669-0.855)	0.312 (-0.025-0.454) p=0.060	0.010 (0.001-0.028) p=0.007
**TMV LeadV4 ≥ 5.0**	**0.767 (0.679-0.857)**	0.284 (-0.039-0.461) p=0.066	0.016 (-0.001-0.053) p=0.060

Established model = sex, age, type 2 diabetes, prior revascularization, CCS class, LV ejection fraction, LV mass index, RR interval, T-wave inversions, QRS duration, Tpe interval and QTc

CI, confidence interval; HR, hazard ratio; IDI, integrated discrimination improvement; LV, left ventricular; NRI, net reclassification index; QTc, corrected QT; TMV, T-wave morphology variations.

Significant differences are indicated in bold.

Table S10: SCD versus non-SCD competing risk regression in ARTEMIS

**Univariate**	**Hazard ratio (95%CI)**
$TMV_1 \geq 2.4$	3.76 (1.80-7.86), $p<0.001$
$TMV_{V4} \geq 5.0$	4.45 (2.27-8.74), $p<0.001$
TMV_1 (per SD)	1.20 (1.03-1.40), $p=0.016$
TMV_{V4} (per SD)	1.32 (1.15-1.51), $p<0.001$
**Multivariate**	
$TMV_1 \geq 2.4$	2.92 (1.51-5.63), $p=0.001$
$TMV_{V4} \geq 5.0$	2.92 (1.51-5.63), $p=0.001$
TMV_1 (per SD)	1.00 (0.80-1.24), $p=1.000$
TMV_{V4} (per SD)	1.21 (1.01-1.45), $p=0.038$

CI = confidence interval. Adjusted for sex, age, type 2 diabetes, prior revascularization, CCS class, LV ejection fraction and LV mass index, RR interval, QRS duration, T-wave inversions, Tpe interval and QTc

Table S11: Association with non-SCD in ARTEMIS

	Univariable		Multivariable	
	**HR (95%CI)**	**P**	**HR (95%CI)**	**P**
RR interval (per 1 SD)	0.651 (0.450-9.42)	0.023	0.740 (0.517-1.059)	0.1
QRS interval (per 1 SD)	1.266 (0.950-1.687)	0.108	-	-
T-wave inversions (any versus none)	2.738 (1.342-5.589)	0.006	-	-
Tpe interval (per 1 SD)	0.867 (0.604-1.243)	0.437	-	-
QTc (per 1 SD)	1.431 (1.035-1.979)	0.03	-	-
TMV Lead I (per 1 SD)	1.417 (1.205-1.667)	<0.001	**1.211 (1.030-1.424)**	**0.021**
TMV Lead V4 (per 1 SD)	1.319 (1.083-1.606)	0.006	1.189 (0.979-1.444)	0.081
TMV Lead I ≥ 2.4	1.478 (0.730-2.988)	0.277	0.983 (0.475-2.034)	0.963
TMV Lead V4 ≥ 5.0	0.740 (0.259-2.116)	0.575	0.681 (0.237-1.955)	0.475

For non-SCD, adjusted for age, type 2 diabetes, prior revascularization, CCS class, LV ejection fraction and LV mass index, RR interval, QRS duration, T-wave inversions, Tpe interval and QTc.

CCS, Canadian Cardiovascular Society grading of angina pectoris; CI, confidence interval; HR, hazard ratio; LV, left ventricular; QTc, corrected QT; SD, standard deviation; SCD, sudden cardiac death; Tpe, T-peak-to-T-end; TMV, T-wave morphology variations.

Table S12: Association with CD in ARTEMIS

	Univariable		Multivariable	
	**HR (95%CI)**	**P**	**HR (95%CI)**	**P**
RR interval (per 1 SD)	0.669 (0.519-0.862)	0.002	0.785 (0.610-1.010)	0.007
QRS interval (per 1 SD)	1.332 (1.102-1.610)	0.003	-	-
T-wave inversions (any versus none)	3.364 (2.033-5.568)	<0.001	2.096 (1.224-3.589)	0.007
Tpe interval (per 1 SD)	0.897 (0.700-1.149)	0.389	-	-
QTc (per 1 SD)	1.605 (1.294-1.992)	<0.001	-	-
TMV Lead I (per 1 SD)	1.327 (1.159-1.521)	<0.001	1.085 (0.929-1.267)	0.301
TMV Lead V4 (per 1 SD)	1.319 (1.151-1.512)	<0.001	1.157 (0.984-1.361)	0.077
TMV Lead I ≥ 2.4	2.356 (1.434-3.869)	0.001	1.352 (0.806-2.269)	0.253
TMV Lead V4 ≥ 5.0	2.215 (1.308-3.751)	0.003	1.421 (0.819-2.465)	0.212

For CD, adjusted for age, type 2 diabetes, prior revascularization, CCS class, LV ejection fraction and LV mass index, RR interval, QRS duration, T-wave inversions Tpe interval and QTc.

CCS, Canadian Cardiovascular Society grading of angina pectoris; CD, cardiac death; CI, confidence interval; HR, hazard ratio; LV, left ventricular; QTc, corrected QT; SD, standard deviation; Tpe, T-peak-to-T-end; TMV, T-wave morphology variations.

Table S13: Association with all-cause mortality in ARTEMIS

	Univariable		Multivariable	
	**HR (95%CI)**	**P**	**HR (95%CI)**	**P**
RR interval (per 1 SD)	0.750 (0.627-0.896)	0.002	-	-
QRS interval (per 1 SD)	1.354 (1.185-1.546)	<0.001	-	-
T-wave inversions (any versus none)	2.162 (1.528-3.058)	<0.001	1.484 (1.025-2.148)	0.037
Tpe interval (per 1 SD)	0.869 (0.728-1.038)	0.121	0,818 (0,681-0,981)	0.03
QTc (per 1 SD)	1.536 (1.315-1.795)	<0.001	1.251 (1,052-1.489)	0.012
TMV Lead I (per 1 SD)	1.213 (1.073-1.372)	0.002	0.991 (0.858-1.144)	0.897
TMV Lead V4 (per 1 SD)	1.190 (1.050-1.349)	0.006	0.980 (0.837-1.148)	0.803
TMV Lead I ≥ 2.4	1.671 (1.181.2.363)	0.004	1.078 (0.748-1.554)	0.686
TMV Lead V4 ≥ 5.0	1.526 (1.014-2.297)	0.043	0.969 (0.624-1.505)	0.890

For ACM, adjusted for age, type 2 diabetes, prior revascularization, CCS class, LV ejection fraction and LV mass index, RR interval, T-wave inversions and QTc.

ACM, all-cause mortality; CCS, Canadian Cardiovascular Society grading of angina pectoris; CI, confidence interval; HR, hazard ratio; LV, left ventricular; QTc, corrected QT; SD, standard deviation; Tpe, T-peak-to-T-end; TMV, T-wave morphology variations.

Figure S1: Normal T-wave morphology references in females for each lead and RR interval value.

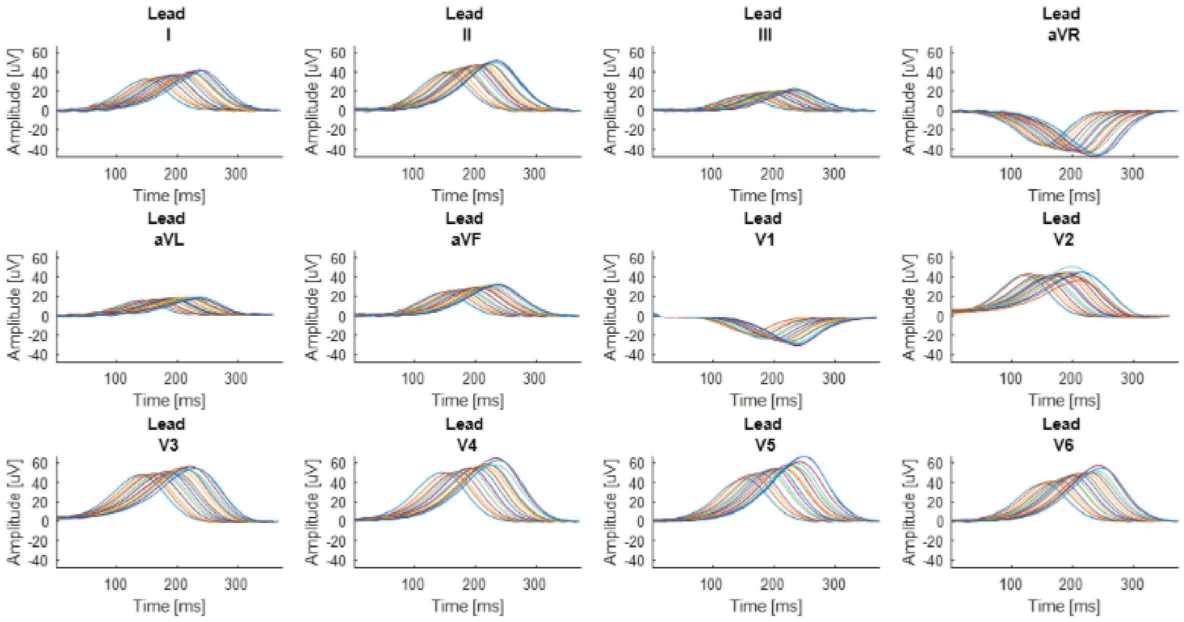

Figure S2: Normal T-wave morphology references in males for each lead and RR interval value.

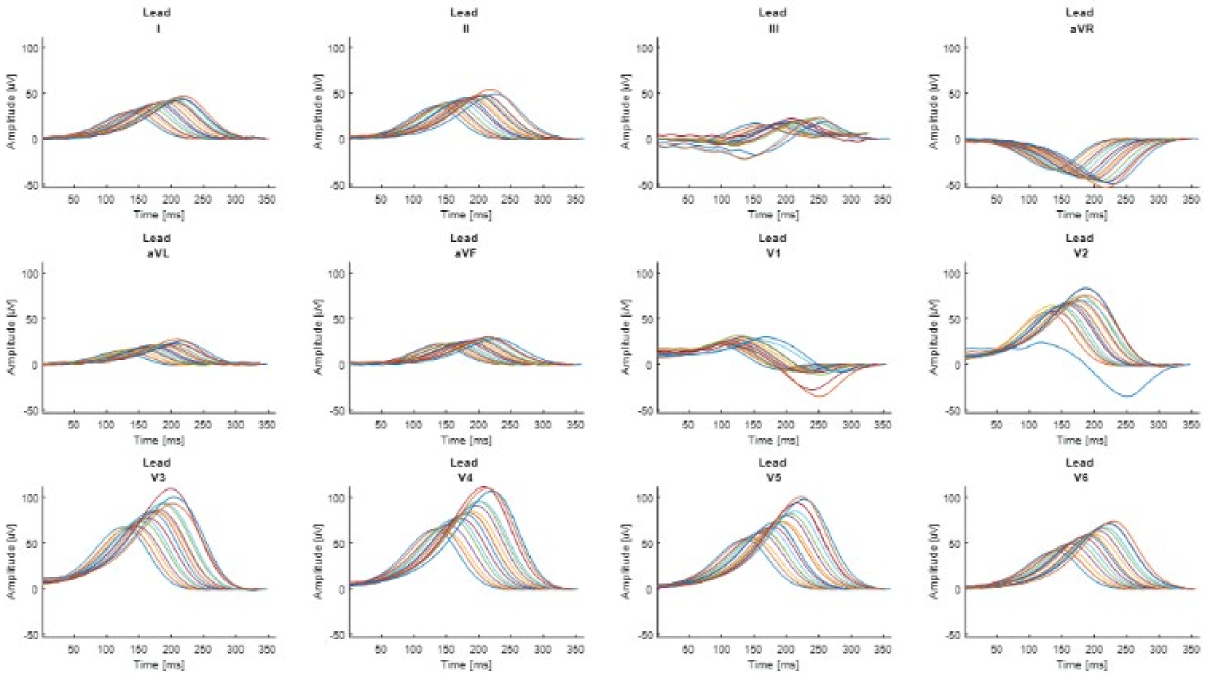

Supplement: Supplementary file 1 — Data S1 Tables S1–S13 Figures S1–S2 References 39, 40, 41 [file JAH3-11-e025897-s001.pdf]
